# Enormous Myoma Pedunculated Through the Cervix: A Surgical Challenge

**DOI:** 10.1155/crog/8044836

**Published:** 2025-07-31

**Authors:** F. Futcher, P. George, N. Habib, P. Von Theobald, A. Birsan-Frances

**Affiliations:** ^1^Department of Obstetrics and Gynecology, North University Hospital of Reunion Island, Saint-Denis, France; ^2^Department of Obstetrics and Gynecology, Hôpitaux du Pays du Mont-Blanc, Sallanches, France; ^3^Department of Obstetrics and Gynecology, Clinique de l'Yvette, Longjumeau, France

**Keywords:** fibroid, pedunculated myoma, vaginal myomectomy

## Abstract

We report the case of a 57-year-old woman who presented to the gynecological emergency department with an enormous 18 cm submucosal leiomyoma pedunculated through the cervix. She was anemic due to bleeding over the past 2 months. The aim of this study was to highlight the challenges of managing such an unusual clinical scenario. We also describe the preoperative and intraoperative methods that can be used to minimize intraoperative blood loss and improve the safety and feasibility of this kind of surgical procedure. In our case, we decided to perform a vaginal myomectomy after a tourniquet on the pedicle to reduce bleeding.

## 1. Introduction

Leiomyomas or uterine fibroids are the most common benign tumors of the female reproductive system. They can affect up to 40% of women [1]. They affect up to nine times more black women than white women. Submucosal myomas, classified as FIGO Type 0, account for around 5% of all uterine myomas [2]. Occasionally, a submucosal leiomyoma may become pedunculated and protrude from the uterine cavity through the cervix and into the vagina. This can cause abnormal uterine bleeding, which can lead to anemia, pelvic pain and heaviness, and the perception of a vaginal mass.

Treatment can be a challenging situation, as management depends on the localization of the myoma, its size, the size of its pedicle and its area of implantation, the patient's desire to preserve her fertility, and the risk of leiomyosarcoma, the suspicious elements of which will be brought to light by a magnetic resonance imaging (MRI). The annual incidence of leiomyosarcomas is approximately 0.4–0.64 per 100,000 women and must be taken into account in the surgical approach [3].

The patient signed a consent form, agreeing to our use of surgical photos, MRI images, and medical information relating to the surgery. She was informed that the documents would be anonymized for publication.

## 2. Case Report

A 57-year-old female patient was admitted to the gynecological emergency department for abnormal vaginal bleeding for over 2 months. She had been in menopause for over 5 years. She also reported pelvic pain and an abdominal mass that had been increasing over the past year.

A reliable medical history was difficult to obtain, as the patient had not seen a doctor in recent years. She had four children, all born by caesarean section. Her last Pap smear was over 20 years ago. She also had a history of high blood pressure, but stopped her treatment with bisoprolol several years before, as she could no longer tolerate it. On admission, her blood pressure was 170/120 mmHg.

Abdominal palpation revealed an abdominal mass extending up to the umbilicus. Gynecological examination revealed a massive lesion of about 20 × 10 cm occupying the entire vagina. During the examination, the cervix was not palpated as we could not even reach the top of the lesion. The lesion was not painful on examination; the walls were smooth and there were no bulging lesions. The lesion was not adherent to the vaginal wall, the walls of the vagina were flexible, and it appeared to not have any invasion of the parametria. There was no bleeding in contact with the lesion and no worrying vascularization.

**Figure 1:**
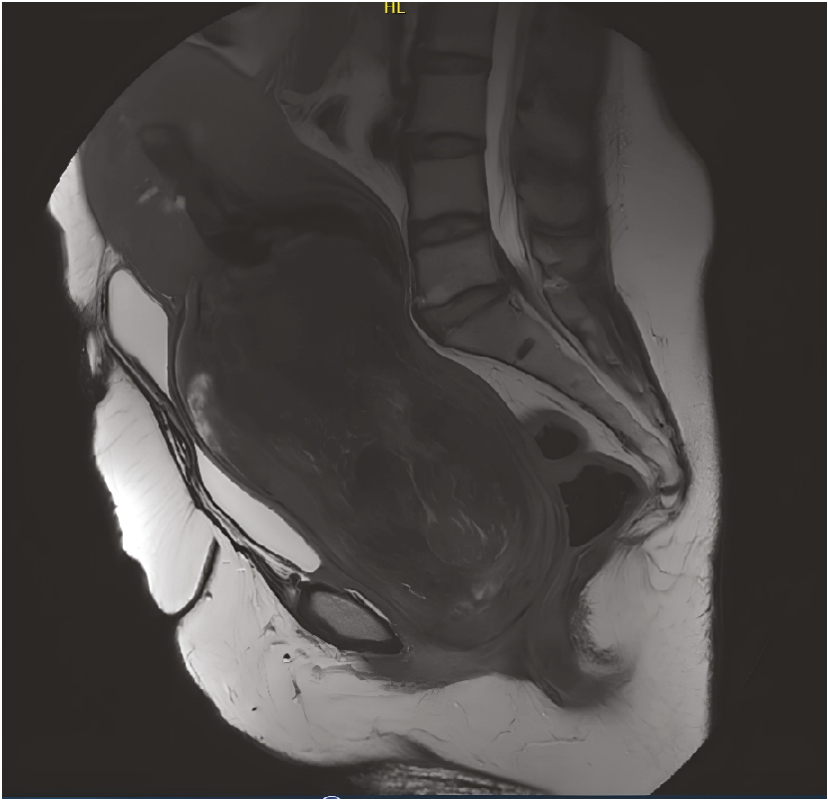
MRI showing pedunculated myoma in the vagina.

**Figure 2:**
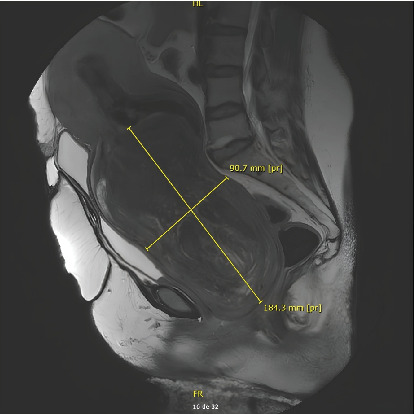
MRI myoma measures.

Ultrasound was inconclusive, so a MRI was ordered. The role of MRI was to assess the nature and morphology of the mass and to describe the stalk and uterine attachment of the prolapsed mass, which could be useful in guiding our treatment. The MRI revealed a mass measuring 18.7 × 11 × 9.5 cm (Figures 1 and 2). The lesion showed some edematocystic remodeling with no diffusion hypersignal or suspicious apparent diffusion coefficient (ADC) area. They suspected a FIGO Stage 0 submucosal myoma with an anterior supraisthmic myometrial insertion pedicle with an overlying cervicouterine hemorrhagic retention. There was also a diffuse thickening of the junctional zone in favor of diffuse adenomyosis. There was a bilateral pyelocaliceal dilatation with bladder reflux.

Her blood hemoglobin level was low, at 7.8 g/dL. The platelet count and the coagulation profile were normal.

Treatment with gonadotropins was not an option, in view of the molecule's action time. Hysteroscopic devascularization was not possible, as the lesion covered the entire vagina, nor was torsion by twisting.

Embolization of the pedicle was not possible due to the large size of the fibroid and the difficult access for our radiology team.

We therefore decided to proceed with a vaginal myomectomy after ligation of the pedicle.

### 2.1. Surgical Technique

Under general anesthesia, the patient was placed in the gynecological position.

**Figure 3:**
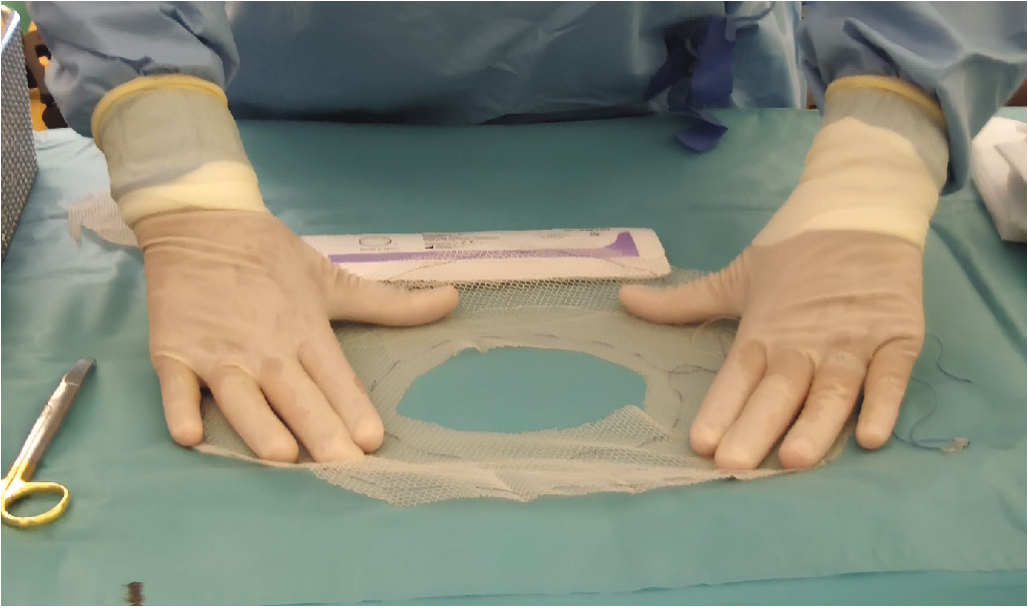
Vicryl mesh preparation.

We used a 30 × 26 cm Johnson–Johnson Vicryl mesh bag (Reference VM 106), the inside of which was cut to the size of the fibroid (Figure 3).

The first option was to use an Endoloop, but the loop was too small and we could not go around the myoma. We then used Bengolea Forceps to push the Vicryl mesh into the vagina around the myoma and up to the myoma pedicle. We then tightened the threads to create a tourniquet. The aim was to reduce bleeding during the myomectomy.

**Figure 4:**
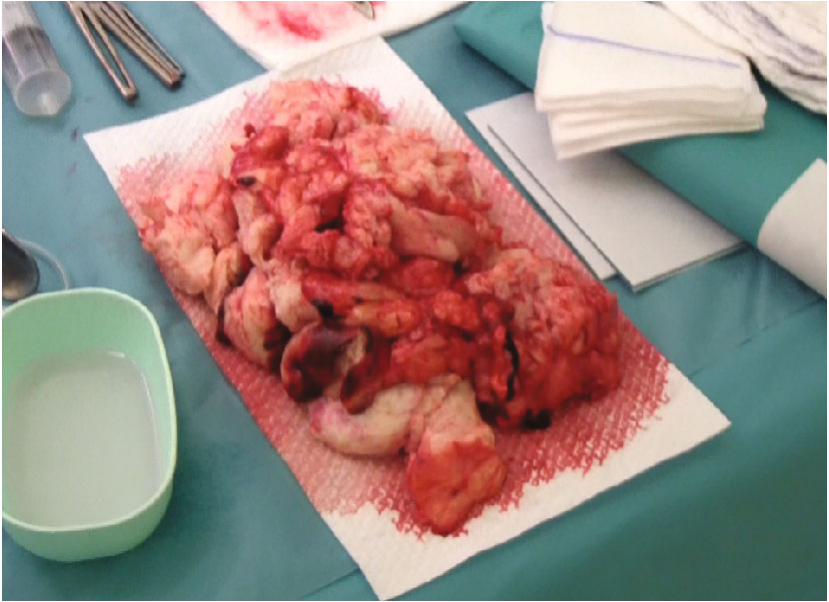
Morcellated myoma.

Using a cold-bladed scalpel, we performed an orange-peel morcellation, gradually reducing the size of the mass. Once the myoma was completely removed (Figure 4), we found a hard-to-reach bleed in the cervix. We placed a Bakri balloon intracervically in order to achieve continuous pressure. The Bakri balloon was inflated with 100 cc of saline water as the cervix was 5 cm dilated. We also added tranexamic acid and 10 units of oxytocin. Blood loss was 500 mL without the need for transfusion.

The Bakri balloon was removed 12 h later without any further bleeding.

The patient was discharged from the hospital the day after the surgery.

One month after the surgery, the patient had no further bleeding or abdominal pain.

The excised tumor weighed 980 g, and pathological examination confirmed that it was a benign myoma.

## 3. Discussion

Leiomyomas or uterine fibroids are the most prevalent benign neoplasm of the female uterus; they have a prevalence of approximately 40% in Caucasian women by the age of 35 years old, with a peak incidence between the ages of 35 and 40 [1]. In France, 8.8% of women present with symptomatic uterine fibroids [4]. The most frequent symptoms are abnormal uterine bleeding (73%), which may occur with or without pelvic pain. About 26% of women experience only pelvic pain. Prevalence can vary according to women's ethnicity. For instance, Black women are reported to have a two to nine times higher prevalence at all ages [5, 6].

Uterine fibroids can be categorized using FIGO staging, based on their location. The most common are the intramural fibroids; they are located within the uterine wall (FIGO 3, 4, and 5). Subserosal fibroids are located near the serosal, on the outer surface of the uterus (FIGO 6 and 7). Finally, submucosal fibroids develop inside or in contact with the uterine cavity (FIGO 1, 2, and 3). Submucosal myomas account for approximately 5% of all uterine myomas [2]. They may be pedunculated into the endometrial cavity, classified as FIGO Type 0 [7], and are attached to the inner wall of the uterus by a stalk [5].

Most prolapsed leiomyomas are small, usually between 1 and 6 cm in diameter, but cases of larger fibroids (over 10 cm) have been reported in the literature [1]. Our case is one of the largest pedunculated fibroids documented to date.

The surgical management of uterine fibroids varies according to their size and location. In the case of externalized pedunculated fibroids, a vaginal approach seemed preferable due to its minimally invasive nature. Using this technique avoids uterine scarring and is less morbid for the patient. This method is advantageous for women who have not completed their family project as it does not impair fertility [2].

We have reviewed the literature in order to outline the different surgical techniques and medical treatments available for the management of prolapsed fibroids.

### 3.1. Embolization

Uterine artery embolization (UAE) is an arteriographic technique designed to infract fibroids by occluding their vascular supply [8].

Spies [8] conducted a retrospective study involving over 3000 patients who underwent UAE and reported a major complications rate of 0.66%. Pain was a frequent complication that often led to prolonged hospitalization, exceeding 48 h. In the first 30 days following the surgery, 2.1% of patients were readmitted for pain management. Also, surgery was required in 1.1% of patients within the first month, with around half of the patients undergoing a transvaginal procedure to treat fibroid expulsion [9]. Another complication of UAE was infection, which has been reported in approximately 1% of embolization procedures [10].

Clinical trials by Prollius et al. showed a significant improvement in many symptoms: a reduction in heavy bleeding symptoms in 86%–92% of patients, pressure effects in 40%–70%, and a substantial reduction in uterine volume (by 50%–60%). However, many studies exclude patients with uterine size greater than 20–24 weeks, which may be due to the fear of an excessive effect of embolization (ischemia, with consequent necrosis and sclerosis) [8, 9].

Pedunculated fibroids may be located either submucosally or subserosally. The ischemic changes induced by embolization may disrupt the peduncle, potentially releasing the fibroid into the peritoneal (in the case of subserosal fibroids) or endometrial (in the case of submucosal fibroids) cavity. The risk of fibroid detachment has led some interventional radiologists to consider pedunculated fibroids as a relative contraindication to UAE. In the case of a subserosal fibroids, the disruption of the stalk may result in fibroid release into the peritoneal cavity. This can potentially lead to chemical peritonitis, resulting in prolonged pain after embolization [11]. In the case of a submucosal pedunculated fibroid, the fibroids can be released through the cervical canal and in some cases through the vagina.

Fibroids, with a stalk diameter less than 50% of the fibroid's maximum width, could present a higher risk of stalk disruption and constitute a relative contraindication to UAE [12]. On the other hand, very large, hypervascular fibroids may respond poorly to embolization, while very small fibroids are unlikely to cause significant symptoms [13].

In our case, embolization was not considered the first line treatment. The interventional radiologist recommended surgical ligation, as the stalk was less than 50% of its size and the fibroid would not be able to be expelled by the vagina on its own, and in any case, a surgical intervention was necessary.

### 3.2. GnRH Analogues

The administration of GnRH analogues significantly reduces the size of fibroids and the uterus by approximately 35%–61% of their initial size [14, 15]. This reduction reaches its maximum after 2–3 months of treatment [14]. Once the analogues are suspended, fibroids return to their original size within 4–10 weeks [14]. GnRH agonists can initially cause a myoma growth due to the flare-up effect, a phenomenon that is not observed with GnRH antagonists. The subcutaneous route is one of the most common modes of administration and is characterized by a high bioavailability with little interindividual variation [15]. GnRH treatment is often used before surgery to facilitate surgical intervention.

However, calcified or fibrous myoma tissue may be unresponsive to hormonal treatment and may therefore be resistant to shrinkage [14].

In our case, this treatment was not an option, as it required too much time before having an effect on the size. The patient was anemic due to heavy bleeding and also had bilateral pyelocaliceal dilatation with bladder reflux.

### 3.3. Cutting of Blood Flow

Some authors recommend clamping the fibroid stalk, if possible, before attempting to remove the myoma [16].

#### 3.3.1. Ligation With Endoloop

Ujihira et al. reported in their study treating 11 patients with prolapsed pedunculated submucosal uterine myoma between July 2009 and 2010 [17]. All patients were treated with an Endoloop ligation of the pedicle, which was accessible through the vagina. Preoperative MRI showed an average tumor size of 30.9 mm, with the largest fibroid measuring 42 mm. The fibroids were left to undergo necrosis. In six patients, the fibroids were expelled spontaneously through the vagina. For the remaining five, the pedicle remained attached, and surgical excision of the necrotic fibroid was necessary. No complications were reported.

Mauri et al. also reported the treatment of a pedunculated fibroid using a loop. The patient presented with a 7 cm mass originating from the internal cervical os, with no palpable base of implantation. The MRI confirmed a leiomyoma with a pedicle attached to the uterine fundus [18]. They proceeded with a loop ligation, which stopped all blood flow, and then proceeded with a vaginal myomectomy.

In our case, the Endoloop was too small, as our mass measured 11 × 9 cm. Sizes reported in the literature do not exceed 7 cm. We therefore used a Vicryl mesh bag, which we cut open to create a loop. We used Bengolea Forceps to position the Vicryl mesh bag up to the pedicle and successfully achieved ligation.

#### 3.3.2. Hysteroscopic Resection of the Stalk

Petros et al. reported the case of a huge, 12 cm pedunculated fibroid, protruding through the cervix into the vagina. A diagnostic hysteroscopy was performed and revealed a fully dilated cervix. Subsequently, the thick fibroid stalk was carefully sectioned using a rigid resectoscope. The hysteroscopy took place in a complete absence of bleeding, with a clear visual field during resection. After sectioning of the large stalk, the fibroid was expelled through the vagina [19].

Hysteroscopic resection of the pedicle was our first choice; unfortunately, the mass filled the entire vagina, preventing advancement of the hysteroscope up to the pedicle.

### 3.4. Bleeding Control

#### 3.4.1. Bakri Balloon

Bakri balloon was the first balloon specifically designed for the uterine cavity and is mainly used for postpartum hemorrhage. The balloon measures 8 cm and can be inflated with up to 800 cc of physiological saline; however, 250–500 cc is the recommended volume used in tamponade practice [20]. Studies have shown a success rate of around 80% in managing postpartum hemorrhage [21]. Bakri balloon has few complications but may include endometriosis (about 5%) or uterine perforation as an immediate complication [22]. For the proper use of the Bakri balloon, the cervix must be dilated; in our case, it was dilated to 5 cm, which facilitated access for the balloon placement.

#### 3.4.2. Chitosan-Covered Gauze

Chitosan-covered gauze (CELOX, from Medtrade Products Ltd, Crewe, United Kingdom) is a 3-m-long hemostatic gauze used to control hemorrhage. Chitosan, a hydrophilic biopolymer obtained by deacetylation of chitin (a major component of the shells of crustaceans such as crab or shrimp) was found to exhibit excellent hemostatic properties. This device can be used in postpartum hemorrhage but also for other pathologies caused by tactical combat injuries [23]. Schmid et al. reported a 75% reduction in hysterectomies after the introduction of chitosan-covered gauze for the control of postpartum hemorrhage compared with no PPH device [24].

Seidel et al. described a case using simultaneously Bakri balloon and CELOX (chitosan-covered gauzes) in 2018. The patient had PPH, losing 2 L of blood due to uterine atony. A chitosan-covered gauze was placed in the uterine cavity, and a Bakri was also placed in the cavity. Hemorrhage stopped after the insertion of both. The balloon was removed after 24 h, and the chitosan-covered gauze was removed after 48 h [25].

#### 3.4.3. Sterile Pad

In the absence of hemostatic devices, sterile compresses can be used for uterine packing. Koffi et al. carried out a case series of 23 patients with uterine fibroids delivered through the cervix. In the event of persistent bleeding, a sterile pad was left in the uterine cavity in contact with the resection area and was removed 24 h after the procedure [6].

In our case, chitosan-covered gauzes were unavailable; we decided to use a Bakri balloon as the pedicle was in the cervix and the cervix was dilated, allowing access. We removed it 12 h after the procedure.

### 3.5. Surgical Approach: What About Abdominal Surgery?

Birsan reported that vaginal myomectomy was associated with shorter operative times and lower morphine consumption compared to laparoscopic myomectomy [26]. The mean operative time was significantly shorter in the vaginal group (96 ± 38 min vs. 166 ± 78 min; *p* < 0.01).

Also, Golan et al. carried out a 10-year retrospective study on vaginal removal of prolapsed pedunculated submucosal myomas. A total of 46 patients were included, 44 of whom underwent vaginal myomectomy. They reported no immediate complications; all patients were discharged the following day [27].

Vaginal myomectomy is generally performed under general anesthesia but can also be performed using local anesthesia [28], which is beneficial in fragile patients to minimize anesthetist exposure [2].

Additionally, vaginal myomectomy reduces the risk of cell dissemination into the abdominal cavity. Uterine sarcoma is rare, with an annual incidence of 0.4–0.64 per 100,000 women. It is estimated that between 1 in 8300 and 1 in 352 women undergoing uterine surgery for suspected fibroids have an unsuspected sarcoma. In such cases, uncontained morcellation of the uterine mass can be associated with the risk of cancer dissemination and potentially worsening of prognosis [3]. In our case, there was no risk of dissemination as the abdominal cavity was not opened.

MRI is the most accurate tool for characterizing uterine masses before surgery [29]. In these large myomas, the MRI also helps to eliminate the risk of leiomyosarcoma. Features such as lobulated or smooth margins, low T2 signal intensity, and low ADC values are signs of benignity.

Vaginal myomectomy is the treatment of choice for prolapsed cervical leiomyoma [27]. It is short, definitive, and in most cases carries minimal disability for the patient.

## 4. Conclusion

Large fibroids can be a challenging surgery. In the case of submucosal fibroids pedunculated through the cervix, we feel that a vaginal approach may be the best approach for the patient. Reducing vascularization either by section, ligation, or embolization may be a good option prior to myomectomy in order to reduce blood loss. Vaginal myomectomy helps also to preserve fertility in women that have not completed their families. It has also been shown to shorten hospital stays and result in less pain.

## Data Availability

Data sharing not applicable to this article as no datasets were generated or analyzed during the current study.
